# Lemierre's Syndrome: A Case Report

**DOI:** 10.7759/cureus.78016

**Published:** 2025-01-26

**Authors:** Tatsunori Shizuku, Naoki Takayama, Ryosuke Yamamuro

**Affiliations:** 1 Internal Medicine, United States Navy Hospital Okinawa, Okinawa, JPN; 2 Transplant Infectious Diseases, Ajmera Transplant Program, University Health Network, Toronto, CAN

**Keywords:** fusobacterium necrophorum, head and neck infection, lemierre's syndrome, septic emboli, septic thrombophlebitis

## Abstract

A 21-year-old woman presented with a sore throat and systemic symptoms, including fever, diarrhea, and chest pain, progressing to sepsis. Imaging revealed a pharyngeal abscess, thrombophlebitis of the left internal jugular vein, and septic emboli. Blood cultures confirmed Fusobacterium necrophorum, and the patient was diagnosed with Lemierre's syndrome. Empirical antibiotics were adjusted to ampicillin-sulbactam based on culture results, leading to a full recovery after a month-long treatment course. This case highlights the diagnostic challenges of Lemierre's syndrome, its severe complications, and the importance of early recognition and targeted antibiotic therapy. Prompt diagnosis and treatment of Lemierre's syndrome are essential to prevent life-threatening complications, underscoring the importance of maintaining a high index of suspicion in patients presenting with systemic symptoms following pharyngitis.

## Introduction

Lemierre's syndrome is a rare but life-threatening condition characterized by septic thrombophlebitis of the internal jugular vein following an initial oropharyngeal infection [1]. Predominantly caused by Fusobacterium necrophorum, this syndrome can lead to severe systemic complications, including septic emboli and organ dysfunction. Early diagnosis and aggressive treatment are crucial for survival. Despite advancements in diagnostics and therapeutics, Lemierre's syndrome remains a diagnostic challenge due to its rarity and non-specific early symptoms [1]. This case report discusses the clinical course, diagnosis, and management of a young patient presenting with Lemierre's syndrome, emphasizing the critical need for early recognition and intervention to prevent fatal outcomes. This case exhibited systemic embolization, which complicated the initial diagnosis. These factors underscore the importance of considering Lemierre's syndrome in differential diagnoses, even when presentations are atypical, to ensure timely and appropriate treatment.

## Case presentation

The patient is a 21-year-old woman with no significant past medical history who presented to our clinic with complaints of a sore throat lasting over a week without dysphagia. The patient reported recent sexual activity with a male partner, including oral and penetrative intercourse. Ten days prior to presentation, she developed odynophagia and coughing. She self-medicated with acetaminophen, which initially relieved her symptoms; therefore, a viral panel was not sent. However, five days prior to admission, she experienced fatigue, high fever, abdominal pain, diarrhea, left-sided chest pain, and a worsening sore throat, accompanied by a loss of appetite. Consequently, she sought care in the emergency department. The patient had a history of a pharyngeal angioma confirmed by imaging studies, but was not taking any medications at the time of presentation. The patient did not have any symptoms related to pharyngeal angioma. On examination, the patient’s vital signs were as follows: blood pressure 95/58 mmHg, heart rate 124 bpm, body temperature 37.6°C, oxygen saturation 98% on room air, and respiratory rate 20/minute. Physical examination revealed pharyngeal erythema, left-sided cervical lymphadenopathy, and tenderness without limitation in mouth opening. Cardiac examination showed no evidence of internal cardiac murmurs or structural abnormalities. Lung auscultation was clear. The abdomen was diffusely tender but without rebound or rigidity. The patient also reported pain in the left thigh. Neurological examination was unremarkable. Initial laboratory investigations revealed a white blood cell count of 7,900/μL and hemoglobin of 13.2 g/dL, with a markedly reduced platelet count of 27,000/μL. Inflammatory markers were elevated, including a C-reactive protein (CRP) level of 32.84 mg/dL. Renal function tests showed a blood urea nitrogen (BUN) of 41.6 mg/dL and creatinine of 1.29 mg/dL (Table 1). Coagulation studies indicated a prothrombin time/international normalized ratio (PT/INR) of 1.14 and an elevated D-dimer of 6.86 μg/mL, consistent with a hypercoagulable state.

**Table 1:** Laboratory results

		(Normal range)
White Blood Cell	7900 /µL	4000-9000 /µL
Red Blood Cell	450 x 10^4 /μL	355-503 ×10^4/μｌ
Hemoglobin	13.2 g/dL	13.0-17.0 g/dL (Male), 11.5-15.5 g/dL (Female)
Platelet	27000 /µL	150,000-450,000 /µL
Neutrophils	92.5 %	40-75 %
Eosinophils	0.6 %	0-6 %
Basophils	0.1 %	0-1 %
Monocytes	1.4 %	2-10 %
Lymphocytes	5.4 %	20-45 %
Creatine Kinase	9 IU/L	30-170 IU/L
Aspartate Aminotransferase	25 IU/L	10-40 IU/L
Alanine Aminotransferase	18 IU/L	7-56 IU/L
Lactate Dehydrogenase	263 IU/L	120-250 IU/L
Gamma-Glutamyl Transpeptidase	67 IU/L	10-50 IU/L (Male), 5-30 IU/L (Female)
Amylase	55 IU/L	30-110 IU/L
Total Protein	6.6 g/dL	6.4-8.3 g/dL
Albumin	3.0 g/dL	3.5-5.0 g/dL
Total Bilirubin	2.5 mg/dL	0.1-1.2 mg/dL
Direct Bilirubin	0.9 mg/dL	0.0-0.3 mg/dL
Blood Urea Nitrogen	41.6 mg/dL	7-20 mg/dL
Creatinine	1.29 mg/dL	0.6-1.2 mg/dL
Estimated Glomerular Filtration Rate	47.3	≥60
Sodium	132 mEq/L	135-145 mEq/L
Potassium	3.0 mEq/L	3.5-5.1 mEq/L
Chloride	94 mEq/L	98-107 mEq/L
Calcium	8.5 mg/dL	8.8-10.2 mg/dL
Zinc	30 µg/dL	60-120 µg/dL
Serum Glucose	132 mg/dL	70-140 mg/dL
C-Reactive Protein	32.84 mg/dL	<0.5 mg/dL
Hemoglobin A1c	5.2 %	<5.7 %
Prothrombin Time-International Normalized Ratio	1.14	0.9-1.2
Activated Partial Thromboplastin Time	33.3 sec	25-35 sec
Fibrinogen Degradation Products	10.2 µg/mL	<5.0 µg/mL
D-dimer	6.86 µg/mL	<0.5 µg/mL
Hepatitis B Surface Antigen	-	-
Hepatitis C Virus Antibody	-	-
Treponema Pallidum Antibody	-	-

**Figure 1:**
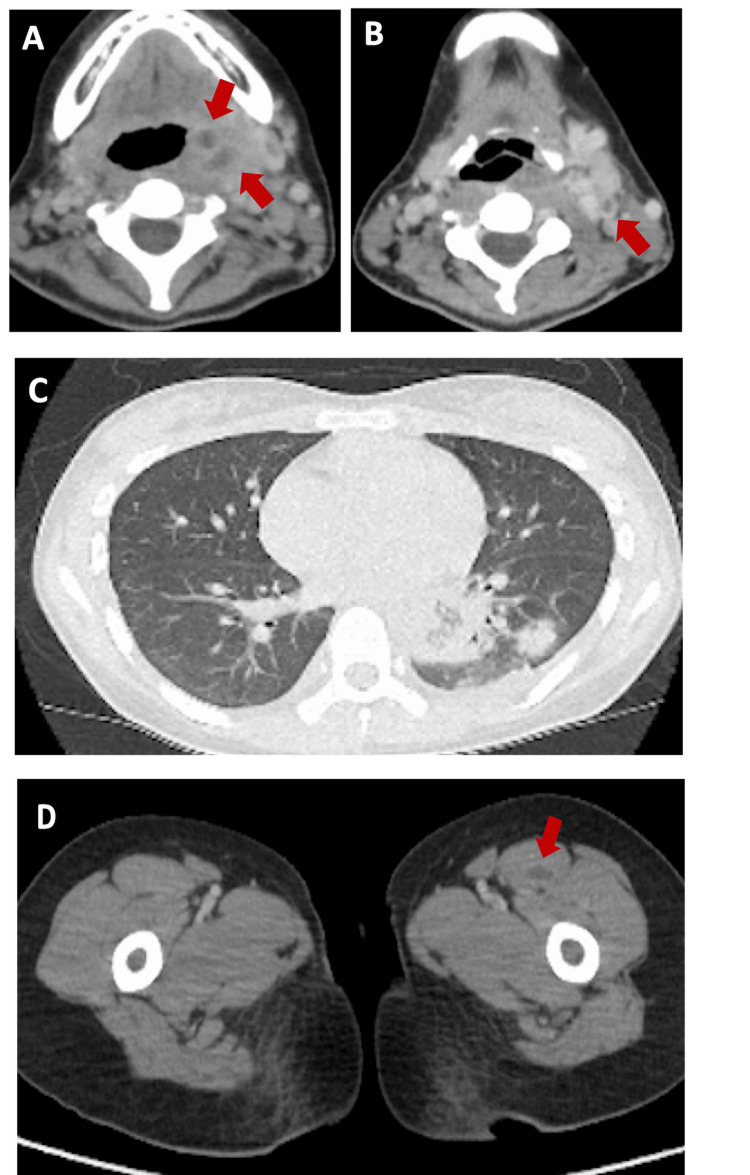
Imaging study A. CT of the neck with contrast shows that two low-density areas with ring enhancement are observed in the left pharyngeal wall, suspected to be abscesses (red arrows: both are approximately 4 mm x 4 mm x 5 mm). B. CT of the neck with contrast indicates that a contrast defect is observed in the left internal jugular vein, with increased surrounding fat tissue density (red arrow). C. CT of the lungs reveals normal bronchovascular bundles and infiltrative shadows, with pulmonary nodules randomly distributed in the bilateral lower lung lobes, without evidence of cavitation D. CT of the thigh with contrast that shows an abscess is observed in the left thigh muscle (red arrow).

Gram staining in urine revealed gram-negative rods, and white blood cell analysis showed a range of 50-99/high power field (HPF). Contrast-enhanced CT imaging revealed a low-density lesion with ring enhancement in the left pharyngeal wall, consistent with an abscess, and lack of contrast enhancement in the left internal jugular vein, indicating thrombophlebitis (Figure 1A, 1B). Surrounding tissue density was increased, and opacities in both lungs suggested septic emboli (Figure 1C). Additionally, an abscess was identified in the left thigh muscles (Figure 1D).

On admission, ceftriaxone 1 g every 24 hours was initiated as empirical treatment for suspected urinary sepsis, targeting Escherichia coli based on the result of the urine analysis. On the second day, blood cultures grew anaerobic gram-negative rods only in anaerobic bottles, confirming bacteremia. The antibiotic regimen was switched to ampicillin-sulbactam 3 g every eight hours, suspecting Lemierre's syndrome. The patient's symptoms improved significantly over the next several days. By the fifth day, blood cultures confirmed Fusobacterium necrophorum, leading to the final diagnosis of Lemierre's syndrome, a septic thrombophlebitis of the internal jugular vein. Ampicillin-sulbactam was continued for 14 days, after which the patient was switched to amoxicillin-clavulanate for 14 days for a total treatment course of one month. At the one-month follow-up appointment, CT with contrast confirmed the resolution of abscesses in the left thigh and pharyngeal lesion, as well as thrombosis in the left internal jugular vein. The patient recovered fully without complications.

## Discussion

Lemierre's syndrome is a rare but potentially fatal condition characterized by septic thrombophlebitis of the internal jugular vein following a pharyngeal infection. It is most commonly caused by Fusobacterium necrophorum, a gram-negative anaerobe that exists as part of the normal flora in the pharyngeal, gastrointestinal, and urogenital tracts. The incidence is approximately 3.6 cases per 1 million, with a higher prevalence among adolescents and young adults [1]. The hallmark feature of Lemierre's syndrome is septic emboli, most commonly affecting the lungs. Interestingly, the initial upper respiratory symptoms often resolve by the time systemic manifestations, such as sepsis, develop [1]. The culture of Fusobacterium requires time to yield definitive results. Initially, cultures may only indicate the presence of an anaerobic organism, which is later identified as Fusobacterium. This finding is critical for diagnosing this infectious disease. Two primary mechanisms have been proposed in the pathogenesis of Lemierre's syndrome. The first suggests that viral infections such as Epstein-Barr virus may compromise the pharyngeal wall, allowing Fusobacterium to invade and spread hematogenously to the internal jugular vein, although the mechanism is still controversial [2]. Pharyngeal angioma could be a risk factor for hematogenous seeding if it was ulcerated, infected, or previously manipulated, as its vascular nature may facilitate bacterial entry from the oral cavity. Disruption of the oral mucous, such as through oral sex, may facilitate the entry of Fusobacterium species, potentially leading to conditions like Lemierre's syndrome. A case study reported an 18-year-old male who developed Lemierre's syndrome after engaging in oral sex, suggesting a possible link between mucosal disruption during such activities and the onset of the syndrome [3]. However, if it has remained stable without such complications, its role as a risk factor is likely minimal. The second, the anatomical pathway theory, posits that a localized abscess in the pharyngeal wall spreads through the lateral pharyngeal space into the internal jugular vein, leading to thrombophlebitis and subsequent septic embolization [2]. 

Lemierre's syndrome is associated with multiple severe complications. Septic emboli, most commonly affecting the lungs, can lead to respiratory distress, abscess formation, and pneumonia. Systemic infections such as sepsis and septic shock are frequent, posing significant risks of organ failure and requiring immediate intervention. Vascular complications, including thrombophlebitis of the internal jugular vein and disseminated intravascular coagulation (DIC), can result in clotting and bleeding abnormalities [1]. Neurological complications, such as meningitis and brain abscesses, may cause severe neurological deficits. Additionally, the infection may disseminate to other organs, causing abscesses in the liver, spleen, and kidneys, impairing their function [1].

Complications such as empyema and neurological deficits are significant concerns in the management of deep neck infections. The incidence of these complications varies depending on factors such as the underlying etiology, timely diagnosis, and the effectiveness of medical interventions. Studies indicate that empyema, a collection of pus within the pleural cavity, occurs in approximately 2-5% of deep neck infections when left untreated or inadequately managed [4]. Neurological deficits, though less common, can arise due to the spread of infection to adjacent neurovascular structures, leading to conditions such as cranial nerve palsies or spinal cord compression. Early recognition and aggressive management, including broad-spectrum antibiotics and appropriate drainage procedures, are crucial to prevent such complications. The threshold for surgical intervention, such as cervicotomy, is typically determined by factors like airway compromise, failure of conservative treatment, or radiological evidence of abscess formation exceeding 3 cm in size. Timely surgical drainage is often warranted to prevent further morbidity and potential mortality [4]. In Lemierre's syndrome, cervicotomy may be considered in cases of deep neck abscesses, persistent or worsening infection despite antibiotics, and to prevent septic emboli by addressing the infection source [5]. While antibiotics remain the primary treatment, surgical drainage is necessary if complications arise. Decisions should be individualized based on the patient's condition, and a multidisciplinary approach is essential for optimal management.

Empiric therapy should target Fusobacterium necrophorum and oral streptococci, with antibiotics resistant to beta-lactamase production, such as piperacillin-tazobactam, carbapenems (e.g., imipenem, meropenem), or ceftriaxone combined with metronidazole [6]. In cases of hemodynamic instability or suspected methicillin-resistant S. aureus (MRSA), vancomycin should be included. For patients with central nervous system (CNS) involvement, broad-spectrum antibiotics with CNS penetration are recommended. Antibiotic therapy generally lasts at least four weeks, with two weeks of intravenous treatment [7]. Persistent bacteremia or clinical worsening despite appropriate therapy necessitates repeat imaging, antimicrobial susceptibility testing, drainage of purulent collections. Anticoagulation remains controversial and is generally reserved for cases with thrombus progression or unresolved fever or bacteremia after five to seven days of treatment [8]. The duration of anticoagulation is guided by clinical improvement and imaging, with input from a hematologist when needed.

Contrasting this case's presentation and outcome with those documented in the literature highlights its uniqueness. While the typical clinical course of deep neck infections often involves progressive swelling, fever, and localized pain, this case presented with atypical symptoms that initially mimicked virus infection conditions, leading to diagnostic challenges and delayed intervention [9]. This patient responded favorably to an extended course of intravenous antibiotics alone, avoiding the need for surgical drainage. This conservative management approach underscores the importance of individualized treatment strategies and close clinical monitoring. In comparison to similar cases in the literature that often report prolonged hospital stays and recurrent infections, this patient's favorable outcome without surgical intervention sets a precedent for cautious but effective conservative management in select cases [9]. Such distinctions emphasize the importance of tailored therapeutic approaches based on patient-specific factors and evolving clinical responses.

Despite advances in treatment, Lemierre's syndrome carries a notable risk of mortality, particularly if diagnosis and intervention are delayed [1]. This underscores the importance of early recognition and aggressive management to improve outcomes.

## Conclusions

This case underscores the critical importance of promptly recognizing Lemierre's syndrome in young patients presenting with pharyngitis and systemic embolic symptoms, such as fever, respiratory distress, or signs of septic shock. Given its potential to cause rapid and severe complications-including septic emboli, organ abscesses, thrombophlebitis, and neurological deficits-early diagnosis and timely administration of appropriate antibiotics are paramount to reducing mortality and preventing long-term morbidity. Since Lemierre's syndrome is rare, it may be easily overlooked, leading to delayed treatment and an increased risk of fatal outcomes. Therefore, healthcare providers, particularly those in emergency or primary care settings, must maintain a high level of suspicion for this condition when managing patients with sore throat, fever, and systemic illness, especially when there are signs of septic or embolic complications. A swift, comprehensive approach to diagnosis and intervention is essential to optimize patient outcomes and mitigate the risk of serious, life-threatening consequences.
